# Assessing Quality of Life in First- and Second-Generation Immigrant Children and Adolescents; Highlights from the DIATROFI Food Aid and Healthy Nutrition Promotion Program

**DOI:** 10.3390/ijerph20032471

**Published:** 2023-01-30

**Authors:** Dimitrios V. Diamantis, Iliana Stavropoulou, Konstantinos Katsas, Lyndsey Mugford, Athena Linos, Matina Kouvari

**Affiliations:** 1 Institute of Preventive Medicine Environmental and Occupational Health Prolepsis, 15121 Athens, Greece; 2 Medical School, National and Kapodistrian University of Athens, 11527 Athens, Greece; 3 Department of History of Science, Faculty of Arts and Sciences, Harvard College, Cambridge, MA 02138, USA; 4 Department of Nutrition and Dietetics, School of Health Science and Education, Harokopio University, 17676 Athens, Greece; 5 Discipline of Nutrition and Dietetics, Faculty of Health, University of Canberra, Canberra, ACT 2601, Australia; 6 Functional Foods and Nutrition Research (FFNR) Laboratory, University of Canberra, Bruce, ACT 2617, Australia

**Keywords:** immigrant children, quality of life, immigration, physical health, mental health

## Abstract

To compare first- and second-immigrant pediatric populations with a non-immigrant pediatric population in terms of quality-of-life metrics, a cross-sectional analysis using data from the DIATROFI Program was implemented. In total, *n* = 2277 students (mean age: 9(4) years) from public schools in Greece participating in the 2020–2021 school year were analyzed. The students’ immigration status (first-generation/second-generation) was defined as per the standard definition. The students’ health related-quality of life (HRQoL) was assessed using a parental-perceived quality of life questionnaire. The sample included 4.8% first-generation and 21.2% second-generation immigrant students. Compared with non-immigrants, the first-generation immigrant students were more likely to have poor HRQoL (odds ratio (OR) = 2.82; 95% confidence interval (95%CI) = 11.75, 4.53), physical (OR = 1.91; 95%CI = 1.18, 3.10), social (OR = 1.94; 95%CI = 1.16, 3.22) and school function (OR = 2.52; 95%CI = 1.54, 4.13). Similar results were observed for second-generation immigrant students regarding HRQoL (OR = 1.68; 95%CI = 1.28, 2.21), physical (OR = 1.60; 95%CI = 1.23, 2.10) and school function (OR = 2.09; 95%CI = 1.58, 2.77). Children with one parent having a country of origin different that the country of residence had elevated odds of having poor emotional health (OR = 1.19; 95%CI = 0.87, 1.64). The family’s affluency level was interrelated with the connection of poor HRQoL and immigration status. The immigrant students have a poorer quality of life depending on their immigration generation and irrespective of their socioeconomic background.

## 1. Introduction

Children of immigrant families can face profound health inequities [1], often resulting in poorer health and many inequalities, from limited access to quality healthcare, parental employment, housing opportunities and barriers in accessing adequate education, to strict immigration policies [1,2,3,4,5]. Apart from the structural inequalities, the acculturative stress attributed to the need for assimilation to new cultural customs can significantly deteriorate their mental and emotional health, deeming them less resilient [6,7]. A reduced parental availability, combined with low parental income and education, can further undermine immigrant youths’ psychological profile [8,9,10]. Over the last twenty years, the number of immigrants around the globe has increased by more than 100 million, reaching 280 million, of whom one in seven is under the age of 19 years old [11]. Considering that about seven in ten immigrants have lived in their migrated European country for more than ten years [12], the issue of tackling immigrant children’s health issues remains significant.

Recent literature underscores immigrant children’s poor quality of life and health in all three dimensions (i.e., physical, mental and social) [2,13]. However, the topic is quite dynamic, while the previous studies are limited, with various methodological limitations. In particular, most of the studies have a limited sample of immigrant children, do not differentiate the health outcomes between first- and second-generation immigrant children, do not account for the country of origin, or often do not adjust for socioeconomic factors [2,14,15,16]. Accounting for socioeconomic factors is critical, as the aforementioned relationship might result from increased poverty, low income, or low educational attainment across migrant families and not be commensurate with the immigrant generation [17].

Considering the aforementioned gaps in the latest literature, the aim of the present work was to evaluate the health-related quality of life (HRQoL) (hereinafter used interchangeably with quality of life) of first- and second-generation immigrant pediatric population children—as perceived by their parents—estimating the potential differences from the non-immigrant pediatric population, using the sample from the “DIATROFI” Program, implemented in Greece. The DIATROFI Program is a school-based food-aid initiative that provides healthy daily meals to students in Greece, aiming to tackle the rising food insecurity due to economic adversities and promote healthy dietary habits [18]. The specific research hypotheses examined here are as follows: (a) more immigrant students live in areas of low socioeconomic status (SES); (b) immigration status is linked with poorer HRQoL, especially when this is accompanied by low SES; and (c) the parental country of origins mediates the examined association between immigration status and HRQoL.

## 2. Materials and Methods

### 2.1. Study Sample

The DIATROFI Program is a school-based food-aid initiative initiated in 2012, and implemented every school year since then, providing healthy daily meals to students of pre-primary (kindergarten), primary and secondary schools from socially disadvantaged regions in Greece. The aim of the Program is to alleviate students’ food insecurity attributed to economic adversities, as well as to promote healthy dietary habits [18].

The sampling procedure is on the basis of schools belonging to an area of low SES. Postal codes with an average per capita income, as depicted in the taxable income database of the Greek Ministry of Finance, below specific thresholds were considered areas of low SES. Specifically, the schools were categorized into three major regions, Attica, Thessaloniki and the rest of Greece, and different thresholds were set by region, so as to cover approximately 25% of all Greek public schools. After establishing initial contacts with all the schools in low-SES areas, the principals were asked to fill in an application for a participation form. The inclusion criteria were income, and specific information provided in the applications, such as the proportion of students estimated to be food insecure by the principals, special characteristics of schools (i.e., students from social institutions and social housing premises, Roma students, etc.), while personal interviews with teachers, parents and other personnel were conducted by an expert in qualitative methodology, to weigh the level of food insecurity in the school. The inclusion of a school in the Program was dependent on the available resources, as well as other administrative matters.

The current work is a cross-sectional analysis using the baseline data of the DIATROFI Program corresponding to the school year 2020–2021. Overall, *n* = 103 schools of *n* = 4870 students from four regions in Greece (i.e., Attica, Macedonia, Central Greece and Thrace) participated in the DIATROFI Program. The data were obtained from *n* = 2151 students (44.2% questionnaire response rate).

### 2.2. Bioethics

The DIATROFI Program was approved by the Bioethics Committee of the National and Kapodistrian University of Athens and the Ethical Committee of Prolepsis Institute (13416–10/2021). The Program is conducted under the auspices of the Greek Ministry of Education and Religious Affairs and was carried out in accordance with the Declaration of Helsinki. All the questionnaires were anonymous and informed consent was obtained from all the parents prior to questionnaire completion.

### 2.3. Baseline Assessment

At the beginning of the school year, anonymized questionnaires are distributed to all schools recruited to participate in the DIATROFI Program. Each questionnaire corresponds to one student and it is answered by the student’s mother or father. In cases where the parents are not able to answer the questionnaire, another guardian is asked to respond.

#### 2.3.1. Sociodemographic Characteristics of Students and Their Families

The baseline assessment included, among other things, the parent’s and student’s age and country of birth, parental education and employment status, family structure (e.g., number of children in the family) and the SES of the student’s family. The parental employment status was organized into three groups: both unemployed parents, one parent unemployed, or both parents employed. The SES of the student’s family was assessed using the Family Affluence Scale (FAS) [19]. This scale comprises four items, and a composite score is assessed for each family. Due to the lack of national thresholds, a three-point ordinal scale was utilized, representing three categories: low- (0–2), moderate- (3–5) and (6–9) high-affluence corresponding to low-, moderate- and high-SES. For the purposes of the present work, the moderate and high family SES groups were merged into one group (moderate/high family SES) to be examined against the low family SES group. The parental educational status was defined as low in cases of 9 or fewer years of education, moderate in cases of 10–12 years of education and high in cases of more than 12 years of education.

#### 2.3.2. Definition of Student’s Immigration Status

The students were categorized into three groups according to the reported parents and student’s country of birth, as follows: first-generation immigrant students, second-generation immigrant students and non-immigrant students. The students born abroad, with at least one parent born abroad, were classified as first-generation immigrants. The students born in Greece, with at least one parent born abroad, were classified as second-generation immigrants. The students born in Greece, with both parents born in Greece, were categorized as non-immigrant students. The reported paternal and maternal country of birth was also used to organize students in groups according to their parents’ origin in relation to their country of residence (Greece) as follows: country of origin same as the country of residence vs. the country of origin different from the country of residence for one parent or both parents.

#### 2.3.3. Students’ HRQoL

The Pediatric Quality of Life Inventory questionnaire (PedsQL)—validated for the Greek population [20]—was used to evaluate the students’ HRQoL. The PedsQL comprises 23 items categorized into four Generic Core Scales: Physical, Emotional, Social and School function. Each question presents an activity, emotion, or condition that the child may have difficulty completing and has five potential answers ranging from “never an issue” to “almost always an issue”. Each Generic Core Scale (i.e., HRQoL, physical function, emotional function, social function, school function) is scored from 0 to 100, with higher scores indicating better quality of life. A total score is calculated, ranging from 0 to 100. Due to the lack of national thresholds, the quartiles of each score (i.e., HRQoL, physical function, emotional function, social function, school function) were evaluated. The 1st quartile (Q1)—corresponding to the group with lower scoring—was used as the cut-off value. The scores below the Q1 corresponded to poor quality of life.

The PedsQL was evaluated for reliability and fit of the factorial structure in our data, as presented in Supplementary Tables S1 and S2. Pearson correlations between the HRQoL score with its 23 items and its four subscales, as well as the physical, emotional, social, and school functions, with their respective questions, were positive and larger than 0.3 (all *ps* < 0.001). The diagonal Cronbach’s α results scored as excellent in the HRQoL score (α ≥ 0.9) and good for its four subscales (0.9 > α ≥ 0.8), thereby confirming the reliability of the PedsQL (Supplementary Tables S1 and S2).

### 2.4. Statistical Analysis

The categorical variables are presented as relative frequencies (%). The continuous variables are presented as the mean values (standard deviation) for the normally distributed variables and the median (interquartile range) for the non-normally distributed variables. The associations between the normally distributed variables and students’ immigration status were evaluated through a one-way analysis of variance. The associations between the non-normally distributed variables and students’ immigration status were evaluated through the Kruskal–Wallis test. Whether these variables were normally distributed was tested through a P-P plot and the equality of variances through Levene’s test. The Bonferroni correction for post hoc analyses was performed. The associations between the categorical variables and students’ immigration status were evaluated through a Chi-square test. The odds ratios (OR) and their corresponding 95% confidence intervals (95%CI) for the immigration status in relation to the examined quality of life endpoints (i.e., poor HRQoL (yes/no); poor physical function (yes/no); poor emotional function (yes/no); poor social function (yes/no); poor school function (yes/no)) were evaluated through multi-adjusted logistic regression analysis. The interaction of immigration status and family SES on quality of life endpoints was examined through logistic regression analysis, including the interaction term “students’ immigration status × SES”. Subsequently, the ORs and their corresponding 95%CI of the student’s immigration status were calculated separately for the low and moderate/high family SES groups, following a standard procedure. The ORs and their corresponding 95%CIs for the parental country of origin in relation to the country of residence, using the same quality of life endpoints, were also evaluated through multi-adjusted logistic regression analysis. For the data analysis, the statistical package for social sciences (IBM SPSS, Chicago) version 20.0 was used, and a *p*-value of ≤0.05 was regarded as statistically significant.

## 3. Results

In total, 27.8% of the study sample were immigrants. About one in four immigrant students were first-generation immigrants. The sociodemographic characteristics according to the student’s immigration status are presented in Table 1. The first-generation immigrant students were about 1.5 times more likely to belong to families of low SES, compared with second-generation immigrant students, and more than twice as high in comparison with non-immigrant students. Additionally, more than 70% of immigrant students had at least one parent being unemployed. Within the first-generation immigrant subgroup, one in four students lived in households where both parents were unemployed. In contrast, unemployment in both parents was only observed in only 8% of second-generation immigrant students, similar to the non-immigrant group. Regarding the parental educational level, around 69% and 40% of parents were assigned to the low educational level group in the case of first- and second-generation immigrant students, respectively.

The parent-perceived quality of life measurements of the children and adolescents who participated in the present study are presented in Table 2. Overall, the first-generation immigrant students presented the worst quality of life (i.e., 42.9% assigned to the group of poor HRQoL), followed by the second-generation immigrant students (i.e., 30.7% assigned to the group of poor HRQoL); in contrast, within the non-immigrant group, 22.1% of the students presented poor HRQoL (*p < 0.001*). A similar trend was observed as regards physical function, with around one in three first- and second-generation immigrant students presenting poor physical function. In the case of social function, the first-generation immigrant students presented the worst HRQoL scoring (i.e., 32.6% assigned to the group of poor HRQoL), followed by the second-generation immigrant and non-immigrant students (i.e., 24.5% and 21.1% assigned to the group of poor HRQoL, respectively) (*p* = 0.02). About 38% and 32% of first- and second-generation immigrant students presented poor school function, which was almost twice as high compared with the respective frequency in the non-immigrant subgroup (*p* < 0.001). As regards emotional function, the immigrant students—either of first- or second-generation—presented slightly lower scores compared with non-immigrant students, yet this difference did not reach the level of significance.

In Table 3, a multi-adjusted logistic regression analysis was performed to assess the association between the student’s immigration status and their likelihood to have a poor quality of life. Significant associations were revealed in the crude analysis, and these were retained in most cases, even after adjusting for family SES. In particular, in the fully adjusted models, the first-generation immigrant students were 2.82 times more likely to be assigned to the group of poor HRQoL compared with the non-immigrant students (OR = 2.82, 95%CI = 1.75, 4.53). The respective association was less strong yet still significant in the case of the second-generation immigrant students, with this group presenting a 1.68 times higher likelihood of presenting poor HRQoL compared with the reference group (OR = 1.68, 95%CI = 1.28, 2.21). Further analysis of separate parameters of quality of life revealed that the observed gap between the non-immigrant and immigrant students was more evident in the case of school function, followed by social function and physical function; first-generation immigrant students still presented stronger discrepancies from the reference group compared with their second-generation counterparts. In relation to the non-immigrant students, the odds of either first- or second-generation immigrant students having poor emotional function was consistently higher than one, yet the association did not reach the level of significance in the fully adjusted model (all *ps* > 0.05).

**Figure 1:**
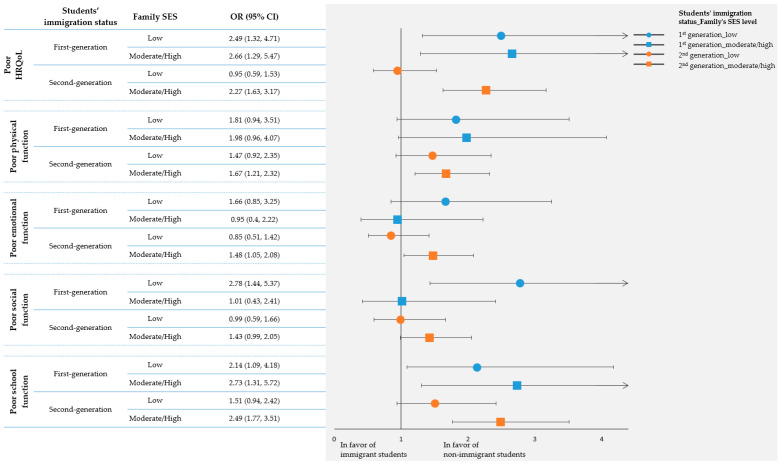
Logistic regression models with interaction analysis to present the association between immigration status and parent-perceived quality of life of students participating in the DIATROFI Program, according to family socioeconomic status. ORs and their corresponding 95%CI were obtained through logistic regression analysis including the interaction terms (students’ immigration status × SES) and using non-immigrant students as reference group. All models were adjusted for age and sex. Abbreviations: Health-related quality of life (HRQoL); socioeconomic status (SES); odds ratio (OR); 95% confidence interval (95%CI).

Among the objectives of the present work was to examine the interaction between students’ immigration status with family SES on students’ quality of life, and then to evaluate the odds of a student having a poor quality of life according to the immigration status as well as the SES of their family. The results are illustrated in Figure 1. Overall, the advantage of a non-immigrant over a first- or second-generation immigrant student was even stronger in the case of families of moderate/high SES. This was more evident in the case of second-generation immigrant students (*p for interaction_(SES × immigration status)_* = 0.003). In specific, within the moderate/high family SES group, second-generation immigrant students had a 2.27 times higher likelihood to present poor HRQoL compared with non-immigrant students; within the low family SES group, no significant association was observed. This trend was retained in all the separate parameters of quality of life, especially within the second-generation immigrant subgroup.

Another research hypothesis investigated here was that the parental country of origin in relation to the country of residence (Greece) is associated with a child’s quality of life. The results are summarized in Table 4. A multi-adjusted logistic regression analysis revealed that the students who had at least one immigrant parent had significantly more chances to present poor HRQoL, as well as inadequate physical, emotional, school and social function. Indicatively, the students with one parent from Greece and one immigrant parent had a 1.9 times higher likelihood to present poor HRQoL compared with students whose parents were native (reference group) (OR = 1.90, 95%CI = 1.27, 2.85). In the case of students with both their parents being immigrants, a similar trend was observed (OR = 1.81, 95%CI = 1.36, 2.42). Focusing on the emotional and social functions, it seems that households with one immigrant parent and the other parent from Greece had almost twice as high a likelihood to have children with impaired social and emotional function compared with the reference group, while, in the case of households with exclusively immigrant parents, no significant association was observed.

## 4. Discussion

The findings of the present work highlight that, within already underprivileged regions, there is a subpopulation that may have additional vulnerabilities, which, in turn, affect their quality of life. In particular, in contrast with the non-immigrant students, the first- and second-generation immigrant students were more likely to belong to families of low SES and parental educational level, and a higher unemployment rate of at least one parent. In addition to and even independently of this socioeconomic gap, immigrant students were more likely to score lower in HRQoL. Further mapping the specific features that contributed to this low HRQoL score—as per students’ immigration status—different patterns were revealed; in particular, first-generation immigration was associated with lower physical, social and school function, while, in the case of second-generation immigration, this was the case only for physical and school function. Maternal and paternal origins seemed to also intervene in the quality of life of their child. Children with one parent born abroad had increased odds of poor emotional and social health, a finding not evident for children with both parents born abroad. This study stands among the very few that examined the quality of life in the immigrant pediatric population, examining the research hypothesis that the specific immigration status may have a more detrimental role as currently discussed in the literature.

The findings presented herein—from an epidemiological perspective—seem to be in line with the official reports from the European Commission and other organizations such as UNICEF [21,22,23]. Overall, the rate of first- and second-generation immigrant children or adolescents was around 50/1000 and 222/1000, respectively. This participant pool may possess an overrepresentation of immigrant children and adolescents compared to the general Greek population [21,22,23], yet this is attributed to the eligibility criteria of the present study with a focus oriented towards areas of low SES, excluding more privileged regions. In addition to this, the present work provides evidence on the socioeconomic profile of immigrant families, revealing that first and second-generation immigrant children and adolescents come from families with fewer sources of income, lower educational levels and less chance of occupation compared to their native counterparts. This is in accordance with country and EU mapping reports that reveal substantially higher rates of poverty and social exclusion in immigrant populations [24,25]. The current study reinforces these results, providing additional evidence according to the immigration status; suggesting that first-generation immigrant children and adolescents belong to families of substantially lower SES, which implies a higher risk of poverty and social exclusion.

Immigrant children and adolescents—especially those who were assigned to the subgroup of first-generation immigrants—presented substantially lower HRQoL compared to their non-immigrant counterparts. The mechanisms through which immigration status affects the quality of life remain complicated and, in some cases, contradictory. In searching through studies with adult populations, we found some of them reveal that low SES, trauma or the stress of migration may decrease overall immigrant health [26], while others suggest the hypothesis of the “healthy immigrant effect”. Focusing on the latter, immigrants tend to be healthier via a selection mechanism [27]. On the other side, in the case of pediatric populations, the hypothesis of the “healthy immigrant effect” is mainly rejected. It has been shown that immigrant child health depends on contextual factors [4,27] and may actually be poorer than native children, an effect exacerbated by strict immigration policies [2,3,4,5,13]. The current study concludes that immigrant children and adolescents display poor HRQoL compared to the native group, further disproving the “healthy immigrant effect” in younger ages and instead identifying a marked health deficit.

As already mentioned, different HRQoL patterns were observed according to students’ immigration status. Immigrant children displayed no difference in emotional function compared to non-immigrant children. It has been shown that migrant mental health levels depend on many factors, including a change in social class upon relocation [28], parent education levels [29], intergenerational conflict and acculturation gap [30], sense of ethnic identity [31], relocation duration [29] and other contextual and/or cultural considerations [4]. These influencing factors have contributed to a lack of clarity in the literature, with some studies claiming that migrant and immigrant children have worse mental health outcomes than native children [2,4,5,32,33], and others presenting neutral associations [27,29]. Yet, if we account for the abridged emotional health of children with only one immigrant parent, our findings emphasize that psychological health effects among immigrant children may be heterogeneous and context-dependent. In contrast to emotional function, social function was reported to be lower in first-generation immigrant children. Social function among immigrant children may be influenced by a variety of factors, most notably the degree of acculturation. Depending on the parental cultural assimilation, children may feel the social strain as they navigate different cultures at home and at school or may face discrimination from their peers [2,4,34]. Our results also indicated that immigrant children with one but not both parents born abroad had worse social health than native children. Immigrant families of first-generation children often reside in areas with other immigrant families from the same area, providing enhanced opportunities for social connectedness and shared experience compared to other environments with fewer immigrant students.

Immigrant children of both generations also displayed decreased school function. Though both immigrant generations can present lower school performance, second-generation immigrant children are often more disadvantaged and have worse performance, due to their late introduction to the national educational system [35]. Second-generation immigrant students have also been shown to have poor school and academic performance and exhibit more negative attitudes toward school than first-generation immigrant children [35,36,37]. The possible explanations include the designation of immigrant children to lower quality school systems [35], first-generation immigrants internalizing home-culture values that emphasize academic achievement [4], or an unequal allocation of parental or teacher support, i.e., providing more proactive support to a first-generation immigrant child than to a second-generation child because they are perceived to be more in need. Nevertheless, our study indicates immigrant students residing in disadvantaged areas are equally as likely to present lower school functioning compared to native children.

Interestingly, while most studies examine only first-generation immigrant children, it appears that second-generation immigrant children may suffer many similar health consequences. It has also been suggested that immigrant children’s health deficits are more prevalent in Northern and Western Europe than in Southern Europe, possibly due to the high proportion of Balkan immigrants to Southern nations compared to predominantly Middle Eastern or African immigrants to other regions [4]. The current study refutes this claim, suggesting that health deficits in immigrant children do appear in Southern Europe, even within a predominantly Balkan immigration population. This finding raises the interesting possibility that immigrant populations may experience health deficits despite similar origins and host cultures. Importantly, the observed health deficits in immigrant children remained after controlling for socioeconomic status, suggesting that poor health outcomes are correlated with immigration status specifically (rather than low affluence in general). The possible causes of worsened health outcomes might include poor home country health status, difficulty in navigating a foreign healthcare system, or physical effects from prolonged toxic stress or travel [26,31].

Another important finding revealed here is the fact that immigration status is related to poorer quality of life irrespective of the general SES. In addition to this, and in contrast with our initial hypothesis, within affluent living conditions, the advantage of native children over their immigrant counterparts seems to be even more apparent and, in some cases, stronger. Several hypotheses can be performed to explain this finding. Low SES is strongly associated with poor HRQoL, attributed to limited access to healthcare services and financial constraints [38]. In this case, the additional aggravating effect of immigration may be lower. On the other hand, in affluent immigrant families, where barriers to health attributed to low affluence are alleviated, we can see the pure—not financially oriented—effect of immigration; principally attributed to the perceived discrimination pertinent to the healthcare service use, language barriers and the need for cultural assimilation [6,7,38].

The current study reveals that both first- and second-generation immigrant children display significant deficits in quality of life accompanied by dysfunctions in school and socialization. Given the substantial increases in immigrant populations within the EU, these results emphasize the importance of interventions to improve immigrant child health. To a limited degree, the global dialogue acknowledges these issues—many interventions address health, nutrition and education within refugee camps and temporary shelters, and the European Commission recently launched an Action Plan on Integration and Inclusion 2021–2017 to support migrants by increasing early educational support, improving health service accessibility and expanding available housing [39]. However, while impactful, these initiatives rarely recognize the ongoing health and socio-economic effects of migration that persist beyond the arrival, the settlement, and even beyond the first generation. This study highlights the urgent need for targeted, long-term investment to address the crisis in immigrant child wellbeing. Because health disparities appear at school age, school-based interventions may prove especially effective for early intervention and improved health outcomes.

### Limitations and Strengths

This study is unique due to its focus on young immigrant children over two generations. The vast majority of immigrant health studies predominantly examine adult or teenage individuals, with little focus on young children in early school systems [40]. Additionally, while first-generation migrants are well studied, immigration’s health effects are poorly characterized in second-generation immigrant children. The current study, therefore, provides novel insights into an area that is understudied compared to most of the existing literature. Nevertheless, several limitations should be reported for a better interpretation of the findings. Firstly, the cross-sectional design of the current analysis prevents establishing causal relations. Secondly, as noted, the sample was not necessarily reflective of the Greek population, as it contained a larger share of immigrant respondents. However, this occurred because the DIATROFI Program primarily targets low-income and high-immigrant neighborhoods. This high proportion of immigrant children also permitted more robust statistical comparisons and therefore strengthened the overall conclusions. Thirdly, sociodemographic data, such as the length of stay and the religious status of immigrant families included in the present study with a potential moderating or mediating effect on the examined associations, were not selected in the DIATROFI Program. Lastly, some students who were classified as “immigrants” may have in fact been refugees who had fled conflict or disaster, as the survey contained no opportunity to specify migration motivation. The trauma and danger of this migration may cause unique health impacts among these students, meriting a unique consideration that is outside the scope of the current study [41].

## 5. Conclusions

This study found that first- and second-generation immigrant children had poorer quality of life than native children, with additional possible effects on mental health, social function, and school engagement. This analysis provides insight into the literature gap on the health challenges of young children in immigrant families, depending on their immigrant generation. Addressing these health disparities in early childhood via targeted, intergenerational school-based interventions is critical to support the health of the recent young migrant wave worldwide.

## Figures and Tables

**Table 1:** Sociodemographic characteristics of students and their families who participated in the DIATROFI Program, in the total sample and according to students’ immigration status.

	Total Sample	Students’ Immigration Status			
Characteristics of Students and Their Family		Non- Immigrant	First- Generation	Second- Generation	*p*-Value
*n*	2151	1553	110	488	
Students’ characteristics					
Students’ age, years	8 (3)	8 (3)	9 (3)	8 (3)	0.085
Boys, %	51.8	51.5	50	52.5	0.892
Student’s highest educational attainment, %					0.002
Pre-primary school (kindergarten)	26.5	25.0	19.4	33.0	
Primary school	65.9	66.7	73.8	61.5	
Secondary school	7.6	8.4	6.8	5.5	
Family characteristics					
Parental employment status, %					<0.001
Both parents are employed	46.0	51.7	21.8	30.7	
One parent is unemployed	46.1	41.3	55.1	61.3	
Both parents are unemployed	7.9	7.0	23.1	8.0	
Paternal educational level, %					<0.001
Low	33.0	27.7	71.1	42.7	
Moderate	41.3	43.3	16.7	40.0	
High	25.7	29.1	12.2	17.3	
Maternal educational level, %					<0.001
Low	25.8	19.1	66.7	39.7	
Moderate	32.7	33.3	21.1	33.3	
High	41.4	47.6	12.2	27.0	
Family socioeconomic status, %					
Low	30.9	25.1	62.0	43.0	
Moderate	54.8	58.1	26.0	50.1	<0.001
High	14.4	16.8	12.0	6.9	
Households with ≥3 underage members, %	28.3	28.9	26.8	26.5	0.595

Parental educational level was defined as low (≤9 years of education), moderate (10–12 years of education) and high (>12 years of education). Family socioeconomic status was defined according to the Family Affluence Scale, i.e., low (FAS = 0–2), middle (FAS = 3–5) and high (FAS = 6–9). Data are presented as mean (standard deviation) for normally distributed continuous variables (age) and % of the corresponding sample for categorical variables. For the normally distributed variables (age), *p*-values were obtained using one-way analysis of variance and the Bonferroni correction for post hoc analysis. For the categorical variables (sex, parental employment status, paternal and maternal educational status, number of underage members in household, family socioeconomic status) a Chi-squared test was performed. Abbreviations: Standard Deviation (SD).

**Table 2:** Parent-perceived quality of life measurements in students participating in the DIATROFI Program.

	Total Sample	Students’ Immigration Status			
Students’ Quality of Life Measurements		Non- Immigrant	First- Generation	Second- Generation	*p*-Value
*n*	2151	1553	110	488	
HRQoL, score (range, 0–100)	91.3 (81–97.8)	91.7 (82.6–97.8)	87.5 (73.8–97.8) *	89.1 (78.3–96.7) *	<0.001
Poor HRQoL, %	25.0	22.1	42.9	30.7	<0.001
Physical function, score (range, 0–100)	96.9 (84.4–100)	96.9 (87.5–100)	90.6 (78.1–100) *	93.8 (81.3–100) *	<0.001
Poor physical function, %	25.6	23.5	34.8	30.7	<0.001
Emotional function, score (range, 0–100)	90 (75–100)	90 (75–100)	87.5 (70–100)	87.5 (70–100)	0.463
Poor emotional function, %	23.0	21.9	26.1	26.1	0.163
Social function, score (range, 0–100)	95 (80–100)	95 (80–100)	90 (70–100)	95 (80–100)	0.055
Poor social function, %	22.4	21.1	32.6	24.5	0.020
School function, score (range, 0–100)	90 (80–100)	95 (80–100)	87.5 (73.8–97.8)	90 (75–100) *	<0.001
Poor school function, %	23.8	20.3	38.5	32.1	<0.001

Students’ quality of life was measured via the Pediatric Quality of Life Inventory questionnaire (PedsQL) answered by students’ parents. For each score (HRQoL, physical function, emotional function, social function, school function), poor quality of life was defined as (score) ≤ Q1(score). Data are presented as median (interquartile range) for quality of life scores since they did not follow normal distribution. *p*-values were obtained using Kruskal–Wallis test. The rest (categorical) variables are presented as % of the corresponding sample, and their *p*-values were obtained through Chi-squared test. Post-hoc analysis was performed using the Bonferroni rule with non-immigrant students as reference group: * *p* < 0.05. Post-hoc analysis was also performed using the Bonferroni rule with first-generation immigrant students as reference group, yet no significant associations were revealed (all *ps* > 0.05). Abbreviations: Health-related quality of life (HRQoL); 1st quartile (Q1).

**Table 3:** Logistic regression models evaluating the association between immigration status and parent-perceived quality of life of students participating in the DIATROFI Program.

	Dependent Variable	Poor HRQoL	Poor Physical Function	Poor Emotional Function	Poor Social Function	Poor School Function	
**Model 1**	**Students’ immigration status**	**OR (95%CI)**	**OR (95%CI)**	**OR (95%CI)**	**OR (95%CI)**	**OR (95%CI)**	**Model** **adjusted for**
	Non-immigrant	*ref*	*ref*	*ref*	*ref*	*ref*	(Crude model)
	First-generation	2.65 (1.72, 4.09) ***	1.74 (1.11, 2.72) **	1.26 (0.78, 2.04)	1.81 (1.15, 2.85) **	2.45 (1.58, 3.82) ***	
	Second-generation	1.57 (1.23, 2.00) ***	1.44 (1.13, 1.84) ***	1.26 (0.98, 1.62) *	1.21 (0.93, 1.57)	1.86 (1.45, 2.37) ***	
**Model 2**	**Students’ immigration status**	**OR (95%CI)**	**OR (95%CI)**	**OR (95%CI)**	**OR (95%CI)**	**OR (95%CI)**	
	Non-immigrant	*ref*	*ref*	*ref*	*ref*	*ref*	Age, sex
	First-generation	2.71 (1.73, 4.24) ***	1.80 (1.14, 2.84) **	1.23 (0.75, 2.02)	1.86 (1.16, 3) **	2.61 (1.64, 4.15) ***	
	Second-generation	1.61 (1.24, 2.10) ***	1.51 (1.17, 1.96) ***	1.17 (0.89, 1.54)	1.23 (0.93, 1.63)	2.05 (1.57, 2.67) ***	
**Model 3**	**Students’ immigration status**	**OR (95%CI)**	**OR (95%CI)**	**OR (95%CI)**	**OR (95%CI)**	**OR (95%CI)**	
	Non-immigrant	*ref*	*ref*	*ref*	*ref*	*ref*	Model 2 + family socioeconomic status
	First-generation	2.82 (1.75, 4.53) ***	1.91 (1.18, 3.10) ***	1.40 (0.84, 2.36)	1.94 (1.16, 3.22) **	2.52 (1.54, 4.13) ***	
	Second-generation	1.68 (1.28, 2.21) ***	1.60 (1.23, 2.10) ***	1.22 (0.92, 1.62)	1.24 (0.92, 1.67)	2.09 (1.58, 2.77) ***	

Students’ quality of life was measured via the Pediatric Quality of Life Inventory questionnaire (PedsQL) answered by students’ parents. For each score (HRQoL, physical function, emotional function, social function, school function), poor quality of life corresponded to (score) ≤ Q1_(score)_ *** *p* < 0.01; ** *p* < 0.05; * *p* < 0.10. Abbreviations: Health-related quality of life (HRQoL); odds ratio (OR); 95% confidence interval (95%CI); 1st quartile (Q1).

**Table 4:** Logistic regression models to evaluate the association between parental country of origin and parent-perceived quality of life of students participating in the DIATROFI Program.

	Dependent Variable	Poor HRQoL	Poor Physical Function	Poor Emotional Function	Poor Social Function	Poor School Function	
**Model 1**	**Parental country of origin in relation to country of residence (Greece)**	**OR (95%CI)**	**OR (95%CI)**	**OR (95%CI)**	**OR (95%CI)**	**OR (95%CI)**	**Model** **adjusted for**
	Same with country of residence (Greece) for both parents	*ref*	*ref*	*ref*	*ref*	*ref*	(Crude model)
	Different from country of residence (Greece) for one parent	1.65 (1.15, 2.37) ***	1.34 (0.93, 1.93)	2.08 (1.46, 2.97) ***	1.55 (1.07, 2.25) **	1.81 (1.25, 2.61) ***	
	Different from country of residence (Greece) for both parents	1.77 (1.37, 2.29) ***	1.58 (1.22, 2.04) ***	0.97 (0.73, 1.29)	1.22 (0.93, 1.61)	1.99 (1.54, 2.58) ***	
**Model 2**	**Parental country of origin in relation to country of residence (Greece)**	**OR (95%CI)**	**OR (95%CI)**	**OR (95%CI)**	**OR (95%CI)**	**OR (95%CI)**	
	Same with country of residence (Greece) for both parents	*ref*	*ref*	*ref*	*ref*	*ref*	Age, sex
	Different from country of residence (Greece) for one parent	1.76 (1.19, 2.62) ***	1.46 (0.99, 2.17) *	1.89 (1.28, 2.78) ***	1.70 (1.13, 2.56) **	2.03 (1.35, 3.04) ***	
	Different from country of residence (Greece) for both parents	1.81 (1.38, 2.37) ***	1.61 (1.23, 2.1) ***	0.94 (0.7, 1.27)	1.24 (0.92, 1.67)	2.19 (1.66, 2.89) ***	
**Model 3**	**Parental country of origin in relation to country of residence (Greece)**	**OR (95%CI)**	**OR (95%CI)**	**OR (95%CI)**	**OR (95%CI)**	**OR (95%CI)**	
	Same with country of residence (Greece) for both parents	*ref*	*ref*	*ref*	*ref*	*ref*	Model 2 + family socioeconomic status
	Different from country of residence (Greece) for one parent	1.90 (1.27, 2.85) ***	1.59 (1.06, 2.38) **	1.91 (1.28, 2.86) ***	1.82 (1.2, 2.77) ***	2.18 (1.44, 3.3) ***	
	Different from country of residence (Greece) for both parents	1.81 (1.36, 2.42) ***	1.66 (1.25, 2.21) ***	1.01 (0.73, 1.38)	1.19 (0.87, 1.64)	2.18 (1.63, 2.93) ***	

Students’ quality of life was measured via the Pediatric Quality of Life Inventory questionnaire (PedsQL) answered by students’ parents. For each score (HRQoL, physical function, emotional function, social function, school function), poor quality of life corresponded to (score) ≤ Q1_(score)._ *** *p* < 0.01; ** *p* < 0.05; * *p* < 0.10. Abbreviations: Health-related quality of life (HRQoL); odds ratio (OR); 95% confidence interval (95%CI); 1st quartile (Q1).

## Data Availability

The data presented in this study are available on request from the corresponding author.

## Supplementary Materials

The following are available online at https://www.mdpi.com/article/10.3390/ijerph20032471/s1, Table S1: Pediatric Quality of Life Inventory questionnaire (PedsQL). Each question of the PedsQL scores from 0: Almost always had problem with… to 100: Never had problem with…; Table S2: Pearson’s correlation and Cronbach’s diagonal results for health-related quality of life (HRQoL) and its subscales with each question; Table S3: Nationality of first-generation immigrant students participating in the DIATROFI Program (N = 110); Table S4: Sociodemographic characteristics of students and their families participated in the DIATROFI Program in the total sample and according to students’ HRQoL.
