# Supplementary material for: Assessing Quality of Life in First- and Second-Generation Immigrant Children and Adolescents; Highlights from the DIATROFI Food Aid and Healthy Nutrition Promotion Program
Source: Int J Environ Res Public Health. 2023 Jan 30;20(3):2471. doi: 10.3390/ijerph20032471 (PMC9915206; doi:10.3390/ijerph20032471)
Supplement: Supplementary file 1 [file ijerph-20-02471-s001.zip › ijerph-2050927-supplementary.pdf]

Supplementary Table S1. Pediatric Quality of Life Inventory questionnaire (PedsQL). Each question of the PedsQL scores from 0: Almost always had problem with… to 100: Never had problem with…

**Physical Functioning Score**	**PF**	**Emotional Functioning Score**	**EF**
1. Walking more than one block	PF1	1. Feeling afraid or scared	EF1
2. Running	PF2	2. Feeling sad or blue	EF2
3. Participating in sports activity or exercise	PF3	3. Feeling angry	EF3
4. Lifting something heavy	PF4	4. Trouble sleeping	EF4
5. Taking a bath or shower by him or herself	PF5	5. Worrying about what will happen to him or her	EF5
6. Doing chores around the house	PF6		
7. Having hurts or aches	PF7		
8. Low energy level	PF8		
**Social Functioning Score**	**SoF**	**School Functioning Score**	**ScF**
1. Getting along with other children	SoF1	1. Paying attention in class	ScF1
2. Other kids not wanting to be his or her friend	SoF2	2. Forgetting things	ScF2
3. Getting teased by other children	SoF3	3. Keeping up with schoolwork	ScF3
4. Not able to do things that other children his or her age can do	SoF4	4. Missing school because of not feeling well	ScF4
5. Keeping up when playing with other children	SoF5	5. Missing school to go to the doctor or hospital	ScF5

Supplementary Table S2. Pearson's correlation and Cronbach's diagonal results for health related-quality of life (HRQoL) and its subscales with each question.

Pearson correlations*	*PF*	*PF1*	*PF2*	*PF3*	*PF4*	*PF5*	*PF6*	*PF7*	*PF8*
PF	1								
PF1	0.7437	1							
PF2	0.7917	0.7649	1						
PF3	0.8029	0.6474	0.7294	1					
PF4	0.7532	0.5068	0.584	0.6213	1				
PF5	0.6993	0.3968	0.397	0.4565	0.3693	1			
PF6	0.7349	0.3833	0.3969	0.4483	0.4499	0.635	1		
PF7	0.5526	0.3172	0.3294	0.3236	0.3672	0.2056	0.3048	1	
PF8	0.6162	0.3505	0.4071	0.4225	0.4551	0.2516	0.3164	0.5057	1
HRQoL score	0.8346	0.5578	0.6268	0.6431	0.608	0.5782	0.624	0.5321	0.5812
Pearson correlations*	*EF*	*EF1*	*EF2*	*EF3*	*EF4*	*EF5*	-	-	-
EF	1								
EF1	0.8107	1							
EF2	0.8402	0.6527	1						
EF3	0.8253	0.5789	0.6772	1					
EF4	0.684	0.4184	0.4207	0.4249	1				
EF5	0.7202	0.5123	0.5028	0.4204	0.4331	1			
HRQoL score	0.7327	0.5606	0.6072	0.5937	0.5567	0.5273			
Pearson correlations*	*SoF*	*SoF1*	*SoF2*	*SoF3*	*SoF4*	*SoF5*	-	-	-
SoF	1								
SoF1	0.7943	1							
SoF2	0.8302	0.6024	1						
SoF3	0.7557	0.4895	0.6784	1					
SoF4	0.8477	0.5235	0.5968	0.4985	1				
SoF5	0.8618	0.6327	0.5682	0.4748	0.7812	1			
HRQoL score	0.8537	0.6977	0.6679	0.6229	0.7389	0.7574			
Pearson correlations*	*ScF*	*ScF1*	*ScF2*	*ScF3*	*ScF4*	*ScF5*	-	-	-
ScF	1								
ScF1	0.8505	1							
ScF2	0.8127	0.6328	1						
ScF3	0.8644	0.8107	0.6667	1					
ScF4	0.6587	0.3495	0.381	0.366	1				
ScF5	0.5586	0.2516	0.2835	0.2503	0.5991	1			
HRQoL score	0.8238	0.7209	0.6725	0.7249	0.5055	0.4571			
	HRQoL score	*PF*	*EF*	*SoF*	*ScF*				
Cronbach's a	**0.926**	**0.863**	**0.836**	**0.876**	**0.809**				

**p<0.001 in all correlations*

Supplementary Table S3. Nationality of first-generation immigrant students participated in the DIATROFI Program (N=110).

	%
**Nationality of first-generation immigrant students, %**	
Balkans	69.7
Other European countries and Russia	11.9
Asian countries	12.8
North & South American and African countries	5.5

Balkans (Albania, Romania, Bulgaria), other European countries, and Russia (Russia, Germany, Ukraine, Switzerland, Cyprus, Italy), Asian countries (Georgia, Syria, Afghanistan, India, Bangladesh, Iran) and North & South American and African countries (Egypt, USA, Canada, Ethiopia).

Supplementary Table S4. Sociodemographic characteristics of students and their family participated in the DIATROFI Program in the total sample and according to students' HRQoL.

	**Total Sample**	**Students' HRQoL**		
**Characteristics of students and their family**		**Poor**	**Good**	***p-value***
N	2,151	538	1,613	
**Students' characteristics**				
Students' age, years	8 (3)	9 (4)	8 (3)	<0.001
Boys, %	51.8	55.4	50.2	0.058
Student's highest educational attainment, %				<0.001
Pre-primary school (kindergarten)	26.5	26.1	27.0	
Primary school	65.9	58.9	67.9	
Secondary school	7.6	15.0	5.1	
**Family characteristics**				
Parental employment status, %				0.001
Both parents are employed	46.0	41.6	47.5	
One parent is unemployed	46.1	46.3	45.5	
Both parents are unemployed	7.9	12.1	7.0	
Paternal educational level, %				0.003
Low	33.0	39.1	30.7	
Moderate	41.3	38.0	42.0	
High	25.7	22.9	27.3	
Maternal educational level, %				<0.001
Low	25.8	34.0	23.9	
Moderate	32.7	31.2	33.1	
High	41.4	34.8	43.0	
Family socioeconomic status, %				0.365
Low	30.9	34.0	31.6	
Moderate	54.8	53.8	53.7	
High	14.4	12.3	14.6	
Households with ≥3 underage members, %	28.3	30.2	28.9	0.596

Parental educational level was defined as low (≤9 years of education), moderate (10-12 years of education) and high (>12 years of education). Family socioeconomic status was defined according to the Family Affluence Scale i.e., low (FAS=0-2), middle (FAS=3-5) and high (FAS=6-9). Students' quality of life was measured via the Pediatric Quality of Life Inventory questionnaire (PedsQL) answered by students' parents. Poor quality of life was defined as (score)≤$Q1_{(score)}$. Data are presented as mean (standard deviation) for normally distributed continuous variables (age) and % of the corresponding sample for categorical variables. For the normally distributed variables (age), p-values were obtained using Student's t-test. For the categorical variables (sex, parental employment status, paternal and maternal educational status, number of underage members in household, family socioeconomic status) chi-squared test was performed. **Abbreviations:** Standard Deviation (SD); Health-related quality of life (HRQoL); 1st quartile (Q1).
